# AMD-Like Substrate Causes Epithelial Mesenchymal Transition in iPSC-Derived Retinal Pigment Epithelial Cells Wild Type but Not *C3*-Knockout

**DOI:** 10.3390/ijms22158183

**Published:** 2021-07-30

**Authors:** Blanca Chinchilla, Rosario Fernandez-Godino

**Affiliations:** The Ocular Genomics Institute at Mass Eye and Ear, Harvard Medical School, Boston, MA 02114, USA; Blanca_chinchilla@meei.harvard.edu

**Keywords:** Bruch’s membrane, AMD, RPE, EMT, complement, collagens

## Abstract

The Bruch’s membrane (BrM) is a five-layered extracellular matrix (ECM) that supports the retinal pigment epithelium (RPE). Normal age-related changes in the BrM may lead to RPE cell damage and ultimately to the onset and progression of age-related macular degeneration (AMD), which is the most common cause of visual loss among the elderly. A role for the complement system in AMD pathology has been established, but the disease mechanisms are poorly understood, which hampers the design of efficient therapies to treat millions of patients. In an effort to identify the mechanisms that lead from normal aging to pathology, we have developed a cell-based model using complement deficient human induced pluripotent stem cell (iPSC)-derived RPE cells cultured on an AMD-like ECM that mimics BrM. The data present evidence that changes in the ECM result in loss of differentiation and promote epithelial mesenchymal transition (EMT) of healthy RPE cells. This pathological process is mediated by complement activation and involves the formation of a randomly oriented collagen meshwork that drives the dedifferentiation of the RPE monolayer. Genetic ablation of complement component 3 has a protective effect against EMT but does not prevent the abnormal deposition of collagens. These findings offer new insights into the sequence of events that initiate AMD and may guide the design of efficient therapies to treat this disease with unmet medical needs.

## 1. Introduction

The retinal pigment epithelium (RPE) sits on a pentalaminar extracellular matrix (ECM) called Bruch’s membrane (BrM). Far from being a mere scaffold, the BrM is an organized structure that provides barrier and filtering functions to the RPE and creates an environment for signal transduction, cell adhesion, proliferation, differentiation, and migration [1,2,3,4]. Changes in the BrM with age include crosslinking of collagen fibers, loss of heparan sulfate proteoglycan, accumulation of advanced glycation end products, and loss of permeability [5,6,7]. Normal age-related changes may influence the onset and progression of diseases such as age-related macular degeneration (AMD), which is the most common type of visual impairment among the elderly [8]. For example, the decreased capacity of exchange between the RPE and the choroid due to a less permeable BrM may affect the ability of these cells to nourish the photoreceptors [9]. Similarly, the loss of elasticity of BrM associated with collagen crosslinking can contribute to its susceptibility to present AMD lesions [6,10,11,12].

Despite the comprehensive research performed to characterize the effects of aging in BrM and how it relates to AMD progression [3,13,14], our understanding of the disease mechanisms is still limited. In order to advance knowledge, a number of in vitro models have been developed that recapitulate some clinical features of the early-stage AMD pathology, including the formation of extracellular deposits called drusen between the RPE and the BrM [15,16,17,18,19]. Our model uses an engineered RPE-secreted ECM that mimics BrM and can be utilized as a substrate to culture RPE cells [16]. Studies using such a platform suggest that ECM signaling can initiate pathological cascades in the RPE, including the activation of the complement system, which is part of the immune system, and the formation of sub-RPE deposits [16]. The results illuminated previous theories founded by clinical and histological studies [16,20,21]. Based on our data and the premise that complement can be activated by spontaneous hydrolysis (tick-over) of complement component 3 (C3) upon contact with surfaces such as the ECM, we believe that changes in BrM with age stimulate complement activation by tick-over in the RPE cells, which promotes their degeneration and disease progression [16,22,23]. Here, we explore how changes in the ECM lead to RPE degeneration and the role of complement in this process.

The transition from early to advanced AMD shares features with the onset of other degenerative diseases associated with chronic inflammation, such as fibrosis [24]. These diseases are characterized by the deposition of ECM fibers that lead to stiffening, overgrowth, and scarring of the tissue [24,25], which in turn lead to alteration of gene expression patterns and inflammatory processes in resident cells [26]. Similarly, the BrM has a strong influence on the fate of the RPE cells, which may lose their differentiation capacity and even die on a damaged BrM [13,27]. Certainly, the difficulty of transplanted RPE cells to attach and survive on a deteriorated BrM has been a major challenge for cell replacement therapies in AMD [13,28]. One hypothesis is that age-related changes in BrM lead to epithelial-mesenchymal transition (EMT) of the resident RPE cells [29,30]. During embryogenesis, the capacity of cells to alternate between epithelial and mesenchymal states is vital [31]. In the context of disease, however, EMT is associated with wound healing, tissue regeneration, and increased production of ECM [32]. In AMD in particular, the RPE cells lose their intercellular junctions and polarity and become more migratory, acquiring an atrophic phenotype [30]. Although this process has been typically described during late stages of AMD, there is evidence that EMT could also contribute to early damage of the RPE [29]. Of note, studies in cancer and other life-threatening human diseases have shown that the EMT process can be regulated by C3 [33,34]. 

In the present study, we have investigated the impact of AMD-like ECM on complement deficient RPE cells. The data show that abnormalities in the ECM cause complement mediated EMT of resident RPE cells, which is prevented by the genetic ablation of C3. These results offer new insights into the mechanisms of disease and may offer a new approach to therapy for AMD.

## 2. Results

### 2.1. Generation of CRISPR-Edited iPSC-RPE Cells C3^KO/KO^

**Figure 1:**
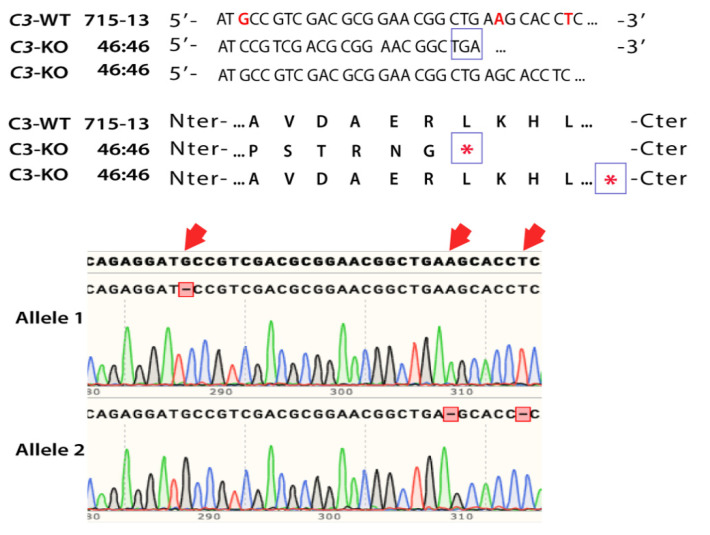
CRISPR edited iPSC-RPE cells *C3*^WT/WT^ and *C3*^KO/KO^. DNA sequence of the iPSC clone 715-13 (*C3*^WT/WT^) and 46:46 (*C3*^KO/KO^). Red bases represent the ones deleted in the knockout clone used for the studies. Sanger sequencing analysis show frameshift deletions (red font, red arrows) in both alleles that result in premature STOP codons (*). The STOP codon in allele 2 is 24 bp downstream of the PAM sequence, but the protein sequence was shortened to simplify the figure. See Supplementary Figure S1.

We had previously published that C3 plays a critical role in the formation of sub-RPE deposits in cell-based models of AMD [15,16,35]. To explore the therapeutic potential of C3, we have generated human induced pluripotent stem cell (iPSC)-derived RPE cells that are knocked out for C3 using CRISPR/Cas9 tools [16,36,37]. Purified Cas9 ribonucleoprotein was complexed with single guide (sg) RNA and transfected into the iPSCs by electroporation. After 48 h post transfection, the cells were lifted and seeded on 6-cm dishes coated with growth factor reduced (GFR) Matrigel at a density of 250 cells/cm^2^ to form clonal colonies. After five to seven days, the colonies were manually transferred to single wells of a 96-well plate and expanded. Among the expanded clones, one had biallelic indels in exon 24, which caused frameshift and STOP codons in each allele (Figure 1). The resultant *C3*-knockout (*C3*^KO/KO^) did not express *C3* (Figure S1). The iPSC clone *C3*^KO/KO^ and one unedited wild type control (*C3*^WT/WT^), were differentiated into RPE following direct differentiation protocols as previously published [37,38,39]. The cells were passaged twice before they were used for further experiments [37,38,39]. 

### 2.2. AMD-Like Substrate Increases Complement Activation by Tick-Over in iPSC-RPE Cell Cultures

The mutation p.R345W in the *EFEMP1* gene causes an inherited macular degeneration clinically similar to AMD [40]. Engineered ARPE19-*EFEMP1*^KI/KI^ cells secrete an AMD-like ECM (aECM) that mimics the BrM of AMD patients [16]. We have shown that normal RPE cells cultured on aECM activate complement and make basal deposits [16]. To investigate if the activation of complement is boosted by hydrolysis of C3 in contact with the aECM [22], we cultured the engineered iPSC-RPE cells on the aECM and measured the amount of C3(H_2_O) in the cell culture media using a recently published method [41]. 

**Figure 2:**
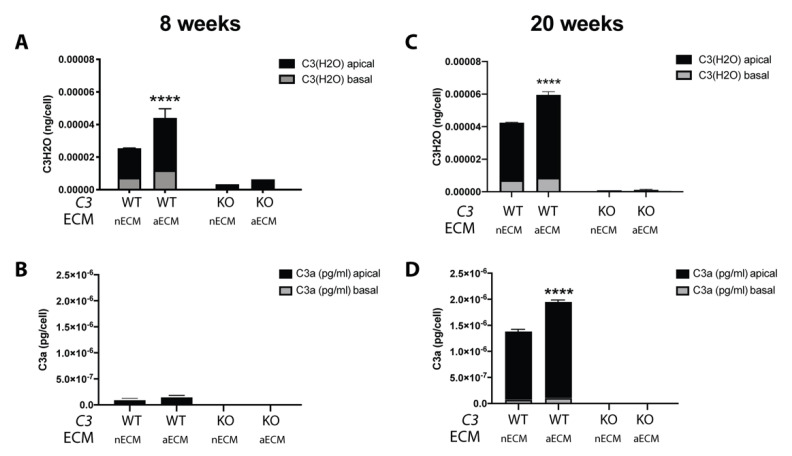
AMD-like substrate increases activation of C3 by hydrolysis. Histograms represent the levels of C3(H_2_O) and C3a measured with ELISA in the apical and basal conditioned media of iPSC-RPE *C3*^WT/WT^ cells and iPSC-RPE *C3*^KO/KO^ cells grown on transwells coated with nECM or aECM for (**A**,**B**) 8 or (**C**,**D**) 20 weeks. Data was normalized to cell counts. (Data represented as mean ± SD. *n* = 4 transwells/type of culture. 2-way ANOVA, **** *p* < 0.0001).

The edited iPSC-RPE *C3*^WT/WT^ and iPSC-RPE *C3*^KO/KO^ cells were seeded onto 12 mm transwells coated with normal ECM (nECM) or aECM [16]. The overall activation of C3 as well as its activation by hydrolysis were measured in the cell culture media at two time points. At eight weeks, increased levels of C3(H_2_O) were already detected in the media of cells cultured on aECM, while the overall activation of the complement system was negligible (Figure 2A,B). At 20 weeks, the elevated levels of C3(H_2_O) were maintained, and increased amounts of C3a were observed in the media of wild type cells grown on aECM compared to the same cells grown on nECM. This result is in line with our observations using primary human RPE cells on human explants of BrM [16]. As expected, C3(H_2_O) and C3a were not detected in the conditioned media of *C3*^KO/KO^ cells. This data corroborated that the activation of C3 was enhanced in the context of the aECM, which can be used as a tool to understand the role of the BrM in the RPE pathology. 

### 2.3. AMD-Like Substrate Causes Dedifferentiation of Mature iPSC-RPE Cells C3^WT/WT^ but Not C3^KO/KO^

**Figure 3:**
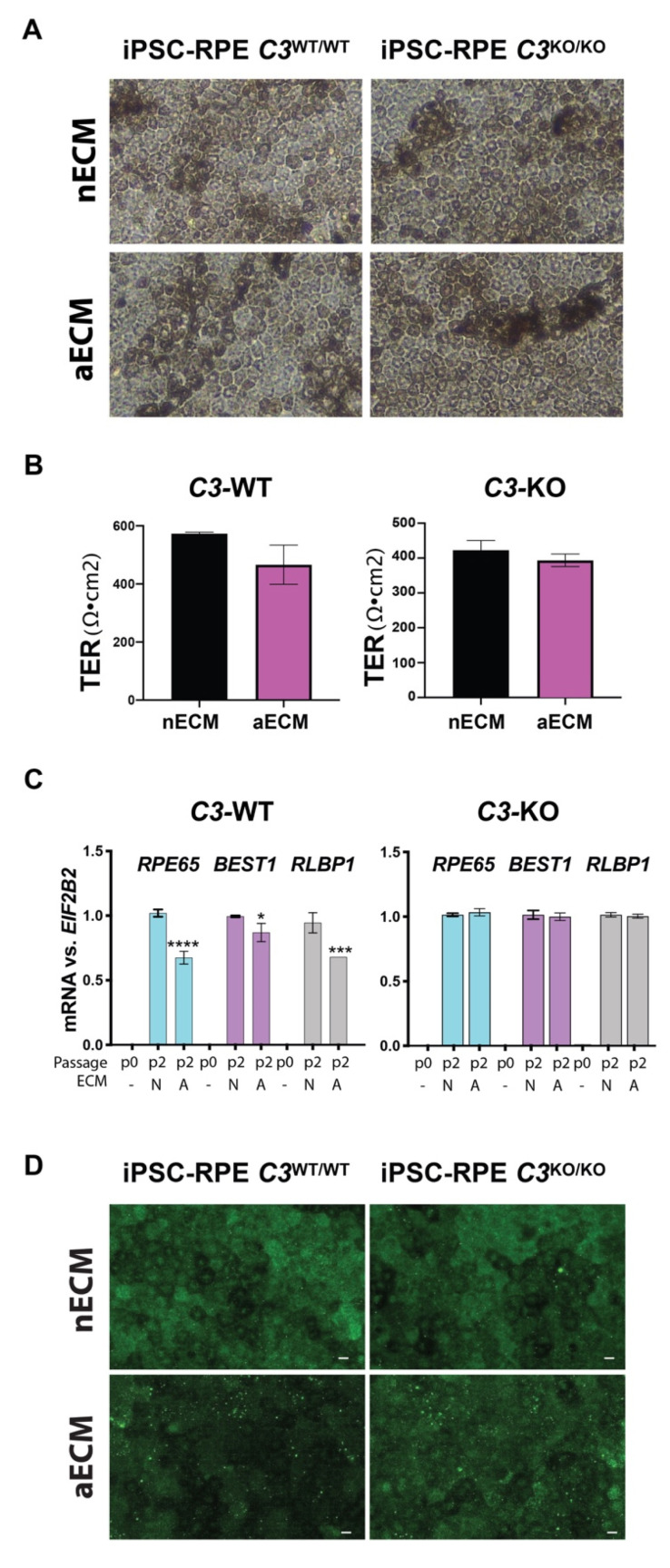
AMD-like substrate causes RPE dedifferentiation. (**A**) Brightfield pictures of mature iPSC-RPE p2 grown for 20 weeks on transwells coated with nECM and aECM. (**B**) TER was measured at 20 weeks in iPSC-RPE *C3*^WT/WT^ and *C3*^KO/KO^ cultured on transwells coated with nECM and aECM. No significant differences were found; the variability observed among cultures falls within the normal range. *n* = 2 transwells on nECM and 4 transwells on aECM of each genotype. (**C**) Histograms represent the mRNA levels of *RPE65*, *BEST1*, and *RLBP1* (normalized to *EIF2B2*) expressed by iPSC-RPE *C3*^WT/WT^ and *C3*^KO/KO^ cells at passage 2 (p2) after 20 weeks in culture. Undifferentiated iPSCs (p0) were used as controls. Data represented as mean ± SD. Two-way ANOVA, **** *p* < 0.0001, *** *p* < 0.001, * *p* < 0.05. *n* = 4 transwells/type of culture). (**D**) Confocal fluorescent pictures of mature iPSC-RPE p2 grown for 20 weeks on transwells coated with nECM and aECM and immunostained with antibodies anti-RPE65. Scale bars: 10 µm.

Next, we explored if the inhibition of *C3* had a protective effect against the RPE pathology caused by changes in the BrM with age. Fully mature iPSC-RPE cells *C3*^WT/WT^ and *C3*^KO/KO^ cells were cultured on nECM and aECM in the absence of serum for a total of 20 weeks in order to allow enough time for the formation of sub-RPE deposits [42]. Around one week post seeding, wild type and mutant RPE cells seeded on nECM proliferated to form a functional monolayer that displayed typical honeycomb morphology and pigmentation, which was maintained throughout the 20 weeks (Figure 3A). The iPSC-RPE cells *C3*^WT/WT^ and *C3*^KO/KO^ seeded on aECM needed around three to five additional days to reach confluence. After that time, all cultures displayed comparable morphology and pigmentation. However, the cell counts remained reduced on the aECM regardless of the genotype (average after 20 weeks: 261,000 cells/transwell coated with nECM vs. 150,000/transwell coated with aECM). The delayed maturation of RPE cells on aECM had an impact on the barrier function of the wild type monolayers, which displayed long-term decreased transepithelial electrical resistance (TER), thereby also decreased permeability compared to cultures on nECM (Figure 3B). Despite lower cell counts, the levels of TER in mutant *C3*^KO/KO^ cultures grown on aECM were maintained, suggesting that C3 was not required for the correct polarization of the RPE monolayer (Figure 3B). 

The expression of typical RPE markers (*RPE65*, *BEST1*, and *RLBP1*) was measured in mature cell cultures by qRT-PCR [39]. As a control, we used undifferentiated iPSC (passage 0), which do not express RPE markers [39]. The RPE markers were significantly decreased in iPSC-RPE cells *C3*^WT/WT^ cultured on aECM (Figure 3C), supporting the premise that the aECM leads to dedifferentiation of the RPE cells. However, differential expression of RPE markers was not found in *C3*^KO/KO^ cells cultured on aECM, which suggests that the absence of C3 may confer advantages to the RPE to differentiate on AMD-like substrates (Figure 3C). This result was validated by immunostaining of the RPE65 marker in all cell types (Figure 3D). Fluorescent images demonstrated that the expression of RPE65 was diminished in wild type cells cultured on aECM, but not in *C3*^KO/KO^ cells (see Figure S2).

### 2.4. AMD-Like Substrate Triggers Epithelial Mesenchymal Transition (EMT) in iPSC-RPE Cell Cultures C3^WT/WT^ but Not C3^KO/KO^

Loss of RPE differentiation has been associated with EMT processes under pathological circumstances, and it is known to play a critical role in AMD [43]. We hypothesized that the loss of RPE markers, the reduced apicobasal polarity of the RPE monolayer and the delayed maturation observed in wild type cells cultured on AMD-like substrate could be associated with EMT processes. To test this hypothesis, we explored the expression of typical EMT markers, including alpha-smooth muscle actin (*αSMA*), vimentin (*VIM*), fibronectin (*FN*) and zonula occludens-1 (*ZO-1*) in fully mature iPSC-RPE cells cultured on nECM and aECM [44]. 

**Figure 4:**
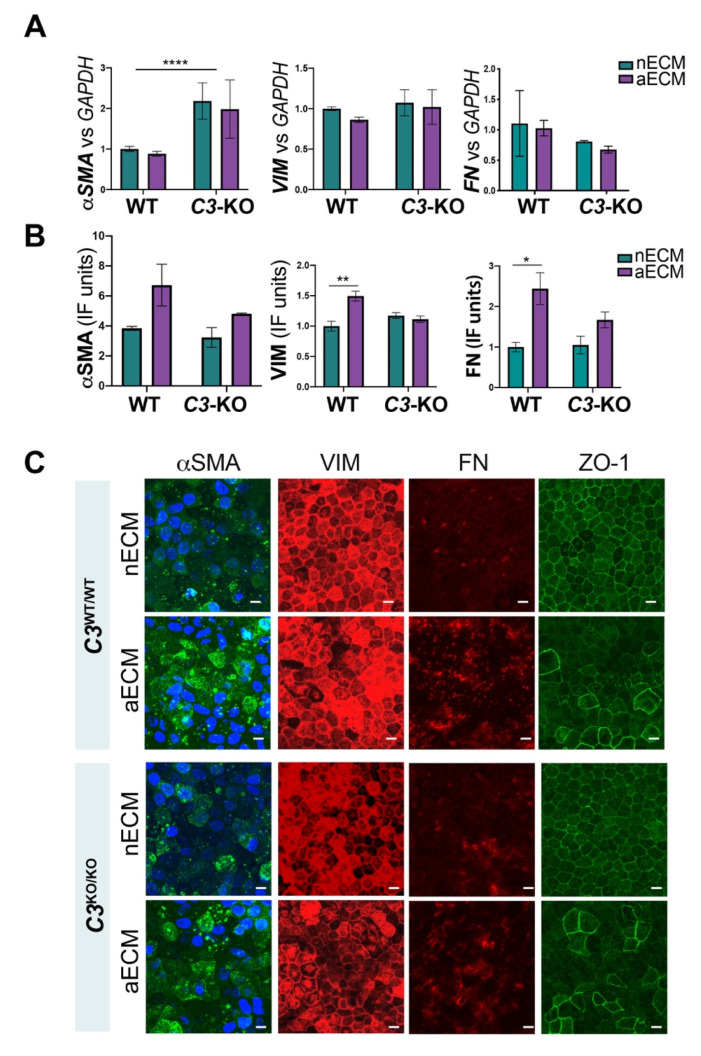
AMD-like substrate triggers EMT in iPSC-RPE cell cultures. (**A**) mRNA levels of *αSMA*, *VIM* and *FN* normalized to *GAPDH* measured in iPSC-RPE cultures *C3*^WT/WT^ and *C3*^KO/KO^ grown on transwells coated with nECM and aECM after 20 weeks. (**B**) Quantification of expression of αSMA, VIM, and FN in the same cultures was carried out combining the fluorescent signal of all z-stacks measured in different areas of the transwell after immunostaining with antibodies. Data was normalized to cell counts. (Data represented as mean ± SD, *n* = 3. 2-way ANOVA, **** *p* < 0.0001, ** *p* < 0.01, * *p* < 0.05). (**C**) Representative confocal fluorescent pictures of iPSC-RPE cultures *C3*^WT/WT^ and *C3*^KO/KO^ grown on transwells coated with nECM or aECM for 20 weeks. Immunostaining with antibodies for *αSMA*, VIM, FN and ZO-1 indicated that wild type and mutant cells cultured on aECM undergo EMT. Scale bars: 10 µm.

The genetic ablation of *C3* led to a two-fold increase expression of *αSMA* (Figure 4A), which is a late marker of EMT [45]. No differences in mRNA levels were observed for *αSMA* between substrates in *C3*^KO/KO^ cultures. Other EMT markers, such as *VIM* and *FN*, did not show significant differences in mRNA expression associated with the ablation of *C3*. Wild type cells showed comparable levels of RNA *αSMA*, *VIM*, and *FN*, regardless of the substrate used for seeding (Figure 4A). 

Despite the unchanged expression of mRNA for the three EMT markers measured, wild type cells cultured on aECM presented increased protein levels of αSMA, VIM and FN (Figure 4B,C). In contrast, immunostaining analyses revealed comparable intensities of EMT markers in mutant RPE cells on nECM and aECM. Yet changes in the staining pattern of αSMA were observed between substrates in both wild type and mutant cultures. Particularly, RPE monolayers on aECM showed irregular expression of αSMA with some cells exhibiting a higher fluorescent signal (Figure 4C). Cell debris also stained intensely, which might indicate that cells undergoing EMT processes are more prone to die (Figure 4B) [30]. 

**Figure 5:**
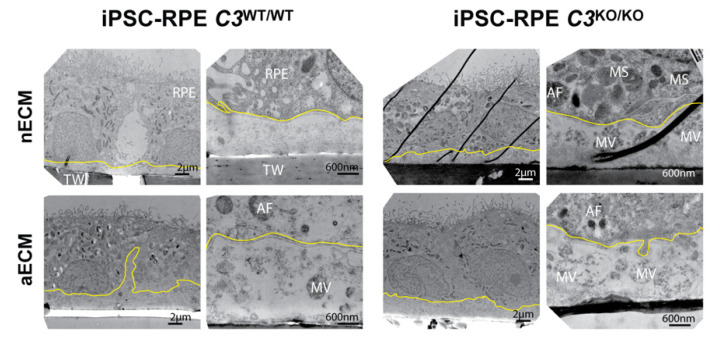
RPE monolayers on aECM show irregular topography and release microvesicles. Transmission electron micrographs of iPSC-RPE cells on transwells coated with nECM and aECM after 20 weeks. Left panels of each genotype are representative low magnification images, and right panels are representative high magnification images. The basal membranes of the RPE are delineated with yellow lines. TW: transwell, MV: microvesicles, MS: melanosomes, and AF: autophagosomes.

One process associated with EMT is the capacity of migration acquired by the cells. Cell migration and redistribution was investigated through the staining pattern of ZO-1, which is typically expressed in the tight junctions between RPE cells and is critical for the homeostasis of the RPE monolayer [46]. ZO-1 presented a singular geographic pattern in wild type and mutant cultures grown on aECM, showing larger disorganized cells that formed an uneven monolayer, of which topography was likely dictated by the structure of the ECM underneath (Figure 4B) [16]. The irregular topography anticipated with the ZO-1 immunostaining in the areas of accumulation of extracellular deposits was confirmed by transmission electron microscopy (TEM) analyses. RPE cultures *C3*^WT/WT^ and *C3*^KO/KO^ grown on AMD-like substrate exhibited some areas along the monolayer in which the extracellular material accumulated underneath caused the topographical elevation of the cells (Figure 5).

The material accumulated underneath the wild type cells was generally comprised of electrodense ECM fibers. Small microvesicles (around 400 nm) could be observed underneath the RPE monolayers on both substrates that became more abundant on aECM (Figure 5). Intracellular accumulation of immature melanosomes and autophagosomes augmented in wild type cells grown on aECM. For the *C3*^KO/KO^ genotype, cells cultured on both nECM and aECM showed extensive intracellular accumulation of autophagosomes and immature melanosomes. A buildup of secreted microvesicles was detected in the extracellular space of mutant cultures as well. Unlike the smaller vesicles observed in the wild type cultures, the size of the microvesicles underneath the mutant cells ranged between 400–800 nm. The abundance of microvesicle mutant cultures increased substantially in the context of AMD-like substrate (Figure 5), which can be associated with augmented cellular stress [47,48].

Signs of loss of RPE differentiation, such as shorter apical microvilli and immature melanosomes, were also noted by TEM analysis in wild type and mutant cultures on aECM (Figure 5, lower magnification pictures). 

### 2.5. AMD-Like Substrate Enhances Deposition of Misaligned and Branched Collagen Fibers

During EMT, cells lose epithelial properties and acquire mesenchymal properties, such as production of MMPs and deposition of ECM, which enhance the emergence of fibrotic processes [49]. Our previous studies have demonstrated a big relevance of collagen IV (*COL IV*) and collagen VI (*COL VI*), as well as matrix metalloproteinase 2 (MMP-2) in the remodeling of the ECM secreted by the RPE [15,16,17]. Here, we investigated the impact of the AMD-like substrate and lack of C3 in this process using iPSC-RPE cells *C3*^WT/WT^ and *C3*^KO/KO^ cultured on nECM and aECM for 20 weeks. 

**Figure 6:**
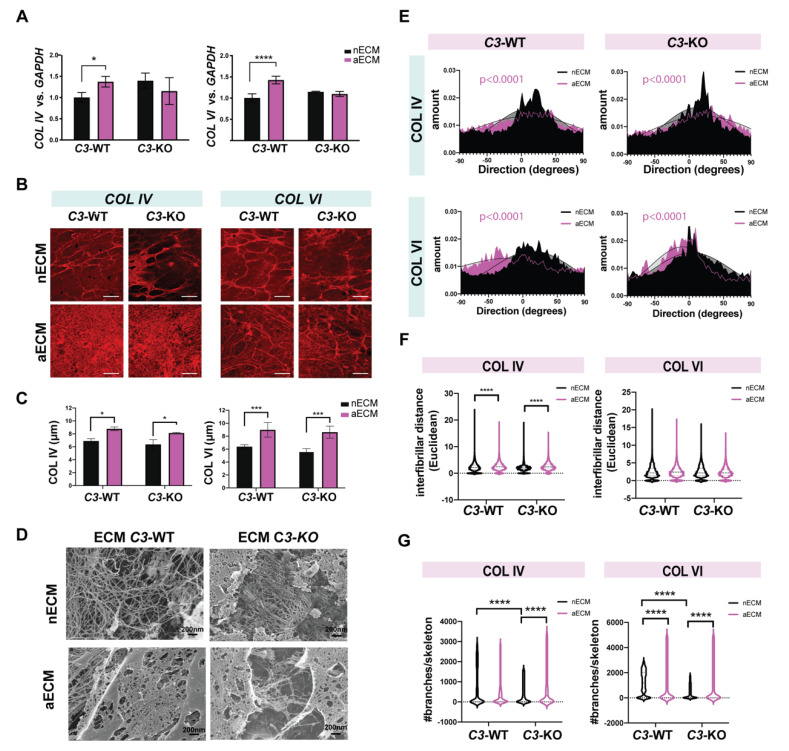
AMD-like substrate impairs the formation of the collagen meshwork. (**A**) mRNA levels of *COL IV* and *COL VI* normalized to *GAPDH* measured in iPSC-RPE cultures *C3*^WT/WT^ and iPSC-RPE *C3*^KO/KO^ grown on transwells coated with nECM and aECM for 20 weeks. (**B**) Representative confocal fluorescent microscopy images: top panels show the expression of collagen IV and collagen VI in iPSC-RPE cultures *C3*^WT/WT^ and iPSC-RPE *C3*^KO/KO^ after 20 weeks on transwells coated with nECM. Bottom panels show the extracellular deposition of COL IV and COL VI in decellularized transwells of iPSC-RPE cells *C3*^WT/WT^ and *C3*^KO/KO^ grown on AMD-like ECM (aECM) for 20 weeks. Scale bar: 25 µm. (**C**) Thickness of collagens layers were measured with confocal using 0.13 µm planes and are plotted in the graphs. Data represented as mean ± SD. *N* = 3 replicates, 2-way ANOVA, * *p* < 0.05, *** *p* < 0.001. (**D**) Scanning electron micrographs of the ECM deposited by iPSC-RPE cells *C3*^WT/WT^ and *C3*^KO/KO^ cultured on nECM and aECM after 20 weeks. (**E**) Directionality of the collagen fibers of representative immunofluorescent images was plotted. Gaussian distributions of cultures on nECM (black) and aECM (pink) were compared using their best fit values in a linear regression and are indicated in the graph with a black line. Distribution was significantly different between substrates for both genotypes (COL IV: *C3*^WT/WT^ nECM vs. *C3*^WT/WT^ on aECM, *p* < 0.0001; *C3*^KO/KO^ nECM vs. *C3*^KO/KO^ on aECM, *p* < 0.0001; COL VI: *C3*^WT/WT^ nECM vs. *C3*^WT/WT^ on aECM, *p* < 0.0001; *C3*^KO/KO^ nECM vs. *C3*^KO/KO^ on aECM, *p* < 0.0001). (**F**) The interfibrillar Euclidean distance (tortuosity) was measured for COL IV and COL VI fibers using FIJI and the BoneJ plugin. (One-way ANOVA–Kruskal–Wallis, **** *p* < 0.0001). (**G**) Violin plots show the branch lengths of collagen skeletons that were defined with BoneJ.

Quantification of collagen transcripts showed that the AMD-like substrate stimulated the production of both *COL IV* and *COL VI* by wild type cells, while no changes were observed in the mutant cultures (Figure 6A). Regarding protein expression, collagens were analyzed intracellularly as well as in the ECM secreted by the RPE. For the first, we did not observe significant differences in expression or localization of COL IV or COL VI along the monolayers. Positive staining was found in the apical side of the cells as well as between the monolayer and the transwell, in the area corresponding to the basal lamina and ECM (Figure S3). A stronger expression was observed for COL VI regardless of the group. This pattern resembled the distribution of collagens found in the sub-RPE deposits in mouse models of AMD [35] and AMD patients [50]. Several observations in our previous work, using ECM made by RPE *EFEMP1*^R345W/R345W^ mutant cells as a substrate to seed fresh RPE cells, have shown that the original ECM is gradually remodeled by the resident cells. 

To gain further understanding of how the AMD-like substrate affects ECM composition, the ECM deposited by wild type and mutant cells grown on normal and AMD-like substrate was immunostained with antibodies for COL IV and COL VI. For this, transwells were decellularized after 20 weeks in culture. Similar expression patterns of COL IV and COL VI were found in the ECM secreted by *C3*^WT/WT^ and *C3*^KO/KO^ cells cultured on transwells coated with normal substrate. In both cases, collagen fibers appeared aligned in an organized mesh with analogous configurations (Figure 6B). The immunostaining pattern changed substantially in both wild type and mutant cultures when the cells were grown on AMD-like substrate, in which case a thicker layer of collagens accumulated underneath the RPE monolayer (Figure 6B,C). In this scenario, both COL IV and COL VI were disposed as a network of entangled crosslinked fibers (Figure 6B), which resembled previous observations using primary human RPE cells [16]. Compared to the robust ECM produced by primary human RPE cells [16], the iPSC-RPE cells produced a thin ECM, which made it difficult to quantify using fluorescence intensity. Instead, the thickness of the collagen layers that filled the space between the RPE monolayer and the transwell was estimated using z-planes obtained with the confocal microscope. This measurement confirmed a thicker deposition of collagens by RPE cells cultured on aECM regardless of their genotype (Figure 6C). In light of these results, C3 did not seem to prevent the deposition of collagens by the RPE on aECM. 

To illustrate the tridimensional structure of the ECM made by both wild type and mutant cultures, we used scanning electron microscopy (SM) (Figure 6D). With this technique, we observed a dense deposition of fibers secreted by the RPE cells *C3*^WT/WT^ grown on the aECM, compared to a finer arrangement of fibers secreted by the same cells grown on nECM (Figure 6D). Mutant cells deposited an entangled network of ECM fibers on both substrates. This finding is in line with other findings in our lab, which suggest that C3 may be necessary for the correct formation of the ECM [17]. 

The spatial orientation of the collagen fibers was evaluated with ImageJ using representative fluorescent micrographs. The directionality of the COL IV and COL VI fibers displayed a Gaussian distribution that changed significantly between substrates (Figure 6E) [51]. The fibers deposited by cells on aECM displayed a flattened Gaussian curve (Figure 6E), which indicated a loss of alignment among fibers on this surface. One way of estimating the tortuosity of the fibers was to measure the interfibrillar distance (Euclidean distance). For this, we built the tridimensional skeleton of representative fluorescent images using the plugin BoneJ of ImageJ [52]. As suggested by the immunostainings and SM, the mesh of intersected fibers deposited by wild type and mutant RPE cells on AMD-like substrate was a randomized disposition of twisted collagen fibers, which resulted in a shortened interfibrillar space (Euclidean distance) (Figure 6F).

Type IV and VI collagens, especially the latest, form a highly branched filamentous meshwork that interacts with other ECM components, conferring biomechanical properties to the BrM [2]. To better characterize the collagen network produced by wild type and mutant RPE cells on different substrates, the COL IV and COL VI fibril branching was measured by skeletonizing immunofluorescence images [52]. The data showed augmented branching of collagen fibers on aECM, remarkably superior for COL VI skeletons. Interestingly, collagen fibers deposited by *C3*^KO/KO^ cells on nECM had less bifurcations than wild type controls (Figure 6G), which would help to prevent the cellular migration associated with EMT. 

### 2.6. AMD-Like Substrate Does Not Alter Collagens Turnover

In the pathogenesis of AMD, a significant role is played by collagens and their regulators, the MMPs [53,54]. Once it was evident that the substrate had an impact on the ECM disposition, we interrogated whether the ECM remodeling was impaired by the aECM. Using zymography, we quantified the activity of MMP-2 in the apical and basal media of iPSC-RPE cultured on transwells coated with nECM and aECM after 20 weeks. 

The activity of pre-processed MMP-2 was similar in wild type and mutant cultures grown on aECM vs. nECM (Figure S4). Processed MMP-2 trended higher in cultures of both genotypes grown on aECM, but the differences were not significant. Accordingly, neither the AMD-like substrate or the ablation of *C3* seem to have an impact on the collagen turnover, unlike previous studies using human fetal RPE cells [16].

## 3. Discussion

The overall aim of this study was to define how changes in the BrM cause the RPE pathology typically observed in AMD, and if this process is mediated by complement activation. Using genome edited iPSC-RPE cells and a modified RPE-derived ECM that mimics BrM, we have developed in vitro models that recapitulate some aspects of the RPE/BrM pathology in AMD. The data demonstrate that the AMD-like substrate leads to dedifferentiation of healthy RPE cells. Second, RPE cells cultured on aECM undergo EMT and increase complement activation by tick-over. Third, AMD-like substrate impairs the formation of the collagen meshwork, which is necessary to sustain the RPE monolayer. Fourth, the genetic ablation of *C3* has a protective effect against the loss of differentiation, and EMT but does not prevent the abnormal deposition of collagens. 

Basement membranes play a key role in epithelial cell function, providing cues for orientation that help to establish and maintain apicobasal polarity and cell differentiation. Thereby, alterations in these membranes should reflect in the health status of the host tissue. Our previous studies have shown that the AMD-like ECM can stimulate complement activation and formation of basal deposits by normal RPE cells, recapitulating clinical features of early/intermediate-stage AMD [16]. The current study determines that such a substrate also perturbs the health of the RPE monolayer, established by the reduced expression of RPE markers in fully mature cells. This, along with the loss of a tight junction barrier similar to that observed in donor tissues [55,56], supports the idea that the early mechanisms of AMD pathology start with changes in BrM. For instance, the irregular topography of the aECM underneath the RPE monolayer promotes its disintegration and facilitates RPE migration, in a similar fashion to the areas with drusenoid deposits in AMD tissues [57]. Another similarity with aged BrM is the limited support of the aECM for RPE cell survival and differentiation [13,27]. Taken together, the data obtained with our model have relevance to the RPE dysfunction observed during the progression of AMD. 

It is important to note that the hydrolysis of C3 was significantly enhanced in the context of aECM, giving weight to the hypothesis that abnormalities in the ECM accelerate the initiation of the complement cascade [16,22]. Besides, the conserved expression of RPE markers and monolayer permeability in *C3*^KO/KO^ cultures indicate that the damage caused to the RPE by the substrate is secondary to the activation of the complement system. An increased complement activity directed by modified substrates has been reported before, which reveals the existence of pathways that connect alterations in the ECM with inflammatory processes in AMD [16,56]. This finding is important to establish the sequence of events that initiates the onset of the disease, which is fundamental to identify the best stage for therapeutic intervention.

The transformation of RPE cells into mesenchymal cells has been described in association with different stages of AMD pathology [29,58]. While a gain in the capacity of migration of the RPE cells has been associated with cellular dysfunction [43], studies using eyes from AMD donors suggest that RPE cells enter EMT to survive the hostile microenvironment during disease progression [30,58,59]. This mechanism of adaptation seems to be mediated by complement, given that the mutant *C3*^KO/KO^ cells did not undergo EMT. This is not the first time that C3 has been recognized as an important mediator of EMT; a similar role for C3 has been described in tumor microenvironments and renal disease [33,34]. Yet C3a can act antagonistically as a suppressor of *αSMA* [60], which explains the increased mRNA levels of this gene observed in *C3*^KO/KO^ cells. This is a promising finding for future therapies since the inhibition of C3 may help to restore the epithelial phenotype and may have the potential to reinstate the functional microenvironment of the RPE/BrM.

Besides its role in maintaining the ECM, collagens can suppress epithelial differentiation and induce EMT, as well as contribute to retinal fibrosis [61,62,63]. This is in line with the finding that cells cultured on aECM enhance the synthesis of *COL IV* and *COL VI* along with the expression of EMT markers. Ultrastructural analysis of collagen architecture revealed disorganized extracellular micro-domains of entangled fibers deposited by RPE cells on aECM, which certainly resemble the collagen crosslinking typically found in BrM with age [64]. The compact ECM secreted by the cells on AMD-like substrate can contribute to decreasing the permeability of the system, which would ultimately lead to the entrapment of debris, proteins, and microvesicles observed in the extracellular space. The increased release of microvesicles is a way of externalizing intracellular proteins, which can contribute to the formation of drusen [48]. 

The excessive deposition of collagens observed on aECM, along with the branching and randomized orientation of the fibers, may represent the fibrotic process that precedes scar formation in dry AMD [65]. The MMP-2 activity was comparable in all cultures, indicating that the accumulation of collagens is a consequence of increased synthesis and deposition, rather than defective turnover. This is different to previous results using short term cultures of human fetal RPE, but it is possible that initial alterations in the ECM turnover are mitigated with time in culture. 

Interestingly, collagen skeletons made by *C3*^KO/KO^ cells on nECM were simpler than wild type controls, displaying fewer branches per skeleton. This would help to prevent the cellular migration and maybe EMT in these cultures. The moderated phenotype in mutant cultures may be also associated with the lack of C3a, which is known to boost collagen synthesis [17,60]. Further analyses must be performed to verify this assumption. 

One limitation of this study is that a single edited clone and its counterpart iPSC WT were used for RPE differentiation and not fully tested for pluripotency. We also acknowledge the limitations of our cell-based model, in which the presence of a mutant structural protein in the ECM (EFEMP1-R345W) leads to changes in the RPE cell biology. Electron micrographs and immunostaining of the decellularized transwells have shown that the ECM secreted by the RPE cells cultured on aECM differs structurally and in composition from the original ARPE19-derived ECM, which was completely remodeled after weeks in culture [16,23]. Thereby, what our model addresses is the effect of abnormalities in BrM on the RPE cell biology. Overall, the data obtained with this model indicate that RPE degeneration is preceded by changes in BrM that act as driving forces of EMT via complement activation. This is a promising finding for the development of efficient therapies that stop disease progression, for instance complement inhibitors could be used to prevent EMT in patients with intermediate AMD. Besides, EMT-targeting therapies could be used to restore the microenvironment to ensure RPE cell survival after transplantation in patients with late-stage AMD.

## 4. Materials and Methods

### 4.1. Clustered Regularly Interspaced Palindromic Repeats (CRISPR)-Cas9 Editing of iPSC-RPE Cells

The induced pluripotent stem cell (iPSC) line IMR90.4 was purchased from WiCell and cultured on growth factor reduced (GFR) Matrigel basement membrane matrix (354230, Corning, NY, USA) coated plates in mTeSR1 (85850, StemCell Technologies, Cambridge, MA, USA) [66,67,68]. The single guide RNAs (sgRNA) used to knock out C3 (5′ CATGATCGGCATGACGCCCA 3′) was designed using the tool E-CRISPR at http://crispr.mit.edu/ (accessed on 10 September 2018) (Boutros lab, E-CRISP-Version 5.4) and synthesized in vitro using the Guide-it sgRNA in vitro transcription and screening system (Takara, Mountain View, CA, USA) following the manufacturer’s instructions. The iPSC at 90% confluence were lifted with TrypLE Express (Gibco, ThermoFisher, Waltham, MA, USA) and resuspended in mTeSR1 with 10 µm of rock inhibitor. Then, 1.5 × 10^5^ cells were resuspended in buffer R and transfected with 450 ng of sgRNA and 2250 ng of Cas9 ribonucleoprotein (electroporation-ready) (Takara, Mountain View, CA, USA) using the neon transfection kit (ThermoFisher, Waltham, MA, USA) programmed at 1100 V during 20 ms, with 2 pulses. Transfected cells were added to 24-well dishes coated with GFR-Matrigel prewarmed with fresh sterile filtered mTeSR1 conditioned media, with rock inhibitor to improve cell viability. Forty-eight hours post-transfection, cells were lifted, strained with a 40 µm cell strainer, and seeded at a confluence of 250 cells/cm^2^ on a 6 cm cell culture dish coated with GFR-Matrigel (low density seeding). Half mTeSR1 and half Fresh sterile filtered conditioned media from iPSC IMR90 combined with 10 µM of rock inhibitor were used during the first 72 h to improve cell viability. Then, mTeSR1 media was replaced daily. After 5 days, 96 colonies were manually picked with a p200 micropipette under a stereomicroscope. Each colony was transferred to a well of a 96-well plate and expanded. DNA was extracted with the QuickExtract DNA solution (Lucigen, Middleton, WI, USA), PCR amplified with the primers F: 5′ CTAACAGTGCAGACCCCCGA 3′ and R: 5′ CAAATGAGGGGAGTGGCTAGG 3′ and sequenced by Sanger. Positive clones were verified by subcloning the PCR amplicons using the TA cloning kit (ThermoFisher, Waltham, MA, USA).

For the differentiation and culture of iPSCs, confluent cultures of iPSC were differentiated using the 14-day direct differentiation protocol previously described [37,38,39]. Briefly, 10 mM of nicotinamide (MilliporeSigma, Burlington, MA, USA), 50 ng/mL of noggin, 10 ng/mL of Dkk1, and 10ng/mL of IGF-1(R&D systems, Minneapolis, MN, USA) were added for days 0–2. From days 2–4, bFGF was added to the cocktail. From days 4–6, cell culture media was enriched with Dkk1, IGF-1, and 100 ng/mL of Activin A (Peprotech, Cranbury, NJ, USA). After day 6, Activin A and SU5402 were added to the media. From days 8–14, an additional 3 µM of CHIR99021 (Stemgent, Cambridge, MA, USA) was incorporated as well. On day 14, non-RPE precursors were manually removed and the enriched premature RPE cultures were seeded on 6-well plates [39]. Cells were passaged with TrypLE after every 30 days and cryopreserved after the second passage, as previously described [39]. Premature iPSC-RPE cells were thawed and seeded onto GFR-Matrigel-coated 6-well plates at a density of 1.5 × 10^5^ cells/cm^2^ and expanded until cultures reached confluence (approx. 5–7 days). Confluent cultures were lifted with TrypLE Express (Gibco, ThermoFisher, Waltham, MA, USA) and plated onto 12-mm transwells (Corning, NY, USA) coated with GFR-Matrigel at a density of 10^5^ cells/cm^2^ in RPE medium* with 5% fetal bovine serum (FBS) [37,69,70]. When the monolayers were confluent (typically 1 week), FBS was removed and the iPSC-RPE cells were cultured in serum-free RPE media for 19 additional weeks [37,69,70].

* RPE medium: 1x N1 Supplement, 1x glutamine, and 1x nonessential amino acid solution, hydrocortisone (20 µg/L), taurine (250 mg/L), and triiodo-thyronine (0.013 µg/L) in alpha MEM + 5% FBS or without FBS [69,70]. 

### 4.2. Transepithelial Electrical Resistance (TER)

Transepithelial electrical resistance (TER) was measured with the EVOM epithelial tissue Voltohmmeter (WPI Inc., Sarasota, FL, USA) as previously described [70].

### 4.3. RNA Expression

RNA was extracted from cell lysates using the RNeasy minikit (Qiagen, Germantown, MD, USA). cDNA was synthesized using the AffinityScript cDNA Synthesis kit (Agilent, Santa Clara, CA, USA). RPE markers *RPE65*, *BEST1*, and *RLBP1* were quantified by quantitative real-time PCR (qRT-PCR) using the TaqMan probes and normalized to *EIF2B2* as previously published [39]. Expression analysis of other genes was performed by qRT-PCR using the following specific primers: *C3* F 5′ AGCGCATTCCGATTGAGGAT 3′ and R 5′ CCTGAGTGCAAGATGACGGT 3′; *aSMA* F 5′ CAACCGGGAGAAAATGACC 3′ and R 5′ CAGTTGTACGTCCAGAGGCATA 3′; *VIM* F 5′ TGCGAGAGAAATTGCAGGA 3′ and R 5′ GTGCCAGAGAAGCATTGTCA 3′; *FN* F 5′ CCATCGCAAACCGCTGCCAT 3′ and R 5′AACACTTCTCAGCTATGGGCTT 3′; *COLIV* F 5′ CTCATTCTGCATCCTGGCTTGA 3′ and R 5′ GCCCTGCTGAGGTCTGTGAACA 3′; *COLVI* F 5′ AATAACGTGGAGCAAGTGTGC 3′ and R 5′ GTCTTCCAGGATCTCCGGC 3′, *GAPDH* F 5′ AGCAAGAGCACAAGAGGAAGAG 3′ and R 5′ GAGCACAGGGTACTTTATTGATGG 3′. 5 ng of cDNA, 200 nM of each primer and 10 µL of x Brilliant III Ultra-Fast SYBR Green (Life Technologies, Grand Island, NY, USA) were combined. Amplification was done in the QuantStudio 3 QPCR system (Thermo Fisher, Waltham, MA, USA) using the following program: 95 °C for 20 s, 40 cycles of 95 °C for 3 s, 60 °C for 30 s followed by a melting curve. 

### 4.4. ELISA

Cell culture media were collected from transwells every 72 h and concentrated to equal volumes through 3 kDa Amicon filters (MilliporeSigma, Burlington, MA, USA). The fraction over 3 kDa was leveled to 200 µL with media. Further, 100 µL was used to quantify C3a using the ELISA kit from Hycult (Hycult, Wayne, PA, USA) (cat #HK354) following the manufacturer’s instructions. C3(H_2_O) was detected in 100 µL of conditioned media using the customized ELISA developed by Elvington et al. [41]. Biological and technical replicates were used. Given that the number of cells was variable in different substrates, concentrations were normalized to cell counts.

### 4.5. AMD-Like Substrate

Gene edited ARPE-19 cells *EFEMP1*^WT/WT^ and *EFEMP1*^KI/KI^ (carrying 2 copies of the mutation C > T c.1034 in the *EFEMP1* gene) were seeded on 12-mm polyester transwells (Corning, NY, USA) at a density of 4 × 10^4^ cells per transwell in DMEM:F12 + 10% FBS. FBS was removed after 72 h. After 4 weeks, transwells were decellularized by incubation with sterile 0.5% Triton X-100 + 20mM NH_4_OH in PBS during 5 min at 37 °C as previously described and washed with PBS thoroughly [16]. The iPSC-RPE cells were seeded immediately on the exposed ECM.

### 4.6. Immunostaining

Decellularized inserts containing exposed ECM were rinsed in PBS, fixed for 10 min in 4% paraformaldehyde (PFA) in PBS, followed by fixation in 1% glutaraldehyde for 30 min at room temperature. Transwell inserts were excised from the chamber, cut in half with a sharp blade and placed on a slide. Samples were permeabilized with 0.1% triton X-100 and blocked with 1% BSA in PBS for 1 h. Primary antibodies RPE65 (AB13826, Abcam, Cambridge, MA, USA), COL IV (AB6586, Abcam, Cambridge, MA, USA) and COL VI (AB6588, Abcam, Cambridge, MA, USA), aSMA (A2547, Sigma-Aldrich, Burlington, MA, USA), VIM (5741, Cell Signaling Technology, Danvers, MA, USA), FN (AB2413, Abcam, Cambridge, MA, USA), and ZO-1 (33-9100, ThermoFisher, Waltham, MA, USA) were diluted 1/100 in blocking buffer and incubated overnight at 4 °C. Secondary antibodies labeled with Alexa-488 or Alexa-555 (Life Technologies, ThermoFisher, Waltham, MA, USA) were incubated at 1/1000 for 1 h at RT. Finally, specimens were incubated with SYTOX (S11380, ThermoFisher, Waltham, MA, USA) for 30 min at RT to exclude the presence of cells, and mounted with Fluoromount G (17984-25, Electron Microscopy Services, Hatfield, PA, USA). Samples were imaged with a TCS SP8 confocal laser scanning microscope (Leica, Allendale, NJ, USA). 

Orthogonal projections: z-stack was built from images taken every 0.13 µm. Tridimensional 90° projections were performed with ImageJ [71]. 

### 4.7. Transmission Electron Microscopy (TEM)

Samples for TEM were prepared as previously described [37,70]. Growth media was removed and membranes fixed in 2.5% glutaraldehyde in 0.1 M sodium cacodylate buffer (pH 7.4) for at least 24 h. Membranes were rinsed out of fixative several times with 0.1 M cacodylate buffer, then post-fixed in 1% osmium tetroxide in 0.1 M cacodylate buffer for 1 h, followed by several rinses again in 0.1 M cacodylate buffer. Membranes were excised from their supports, cut into strips and dehydrated through a graded series of ethanol (30% to 100%), dehydrated briefly in 100% propylene oxide, and allowed to pre-infiltrate at least 2 h in a 2:1 mix of propylene oxide and Eponate resin (Ted Pella, Redding, CA, USA; #18010). Samples were then transferred into a 1:1 mix of propylene oxide and Eponate resin for overnight infiltration at room temperature on a gentle rotator. The following day, specimens were allowed to infiltrate at least 3 h in freshly prepared 100% Eponate resin, then transferred into BEEM capsules (Size 3, Electron Microscopy Sciences, Hatfield, PA, USA; EMS#69910-01) with fresh Eponate resin and placed in a 60 °C oven for resin polymerization (24–48 h). Thin (70 nm) sections were cut using a Leica EM UC7 ultramicrotome, collected onto formvar-coated grids, stained with 2% uranyl acetate and Reynold’s lead citrate and examined in a JEOL JEM 1011 transmission electron microscope at 80 kV. Images were collected using an AMT digital imaging system with proprietary image capture software (Advanced Microscopy Techniques, Danvers, MA, USA).

### 4.8. Scanning Electron Microscopy (SM)

Exposed ECM (from decellularized transwells) were fixed in 4% PFA. Transwell inserts were washed in PBS, hydrated in dH_2_O for 5 min and dehydrated by serial ethanol (35 to 100%) as previously described [16]. Critical dehydration was performed with the SAMDRI-795 system. After dehydration, specimens were coated with Chromium using a Gatan Ion Beam Coater for 10 min and imaged by a Field Emission Scanning Electron Microscope at the Harvard University Center for Nanoscale Systems.

### 4.9. Fiber Orientation

#### 4.9.1. Directionality of Collagen Fibers

Directionality of collagen fibers was calculated using representative confocal fluorescent images placed in the same orientation. Z-stack was performed using the maximum fluorescence, then directionality was analyzed and plotted in histogram starting in −90° and ending in + 90° with ImageJ. Gaussian curves were compared using a linear regression fit and comparing the best fit values between data sets. Null hypothesis: one curve for all data sets. 

#### 4.9.2. Interfibrillar Distance and Branch Information

Interfibrillar distance and branch information were calculated using the same representative confocal images (z-stack) converted to binary images. The BoneJ plugin was used to analyze the skeleton. The Euclidean distance, and branches/skeleton were displayed in the branch information table. Data was sorted from largest to shortest value and plotted in GraphPad prism. 

### 4.10. Zymography

Ten µL of conditioned media concentrated with 3K Amicon filters were loaded onto Novex 10% gelatin gels (Life Technologies, Grand Island, NY, USA). Zymography assays were performed following manufacturer’s instructions. Samples were run at 125 V for 2 h, then renatured for 1 h at RT and developed overnight at 37 °C. Gels were stained with Simply Blue SafeStain (Life Technologies, Grand Island, NY, USA) and scanned using the Odyssey system (Li-Cor, Lincoln, NE, USA). Gelatinase activity was quantified through the intensity of the bands with the software ImageStudioLite (Li-Cor, Lincoln, NE, USA). Since the cell viability diminished considerably on aECM, zymography results were normalized to cell numbers. 

## Data Availability

Not applicable.

## Supplementary Materials

The following are available online at https://www.mdpi.com/article/10.3390/ijms22158183/s1, Figure S1: *C3*-knock out clone details. Figure S2: Quantification of RPE65 immunofluorescence. Figure S3: Intracellular expression of COL IV and COL VI, Figure S4: ECM turnover.
