# Supplementary figures and images for: AMD-Like Substrate Causes Epithelial Mesenchymal Transition in iPSC-Derived Retinal Pigment Epithelial Cells Wild Type but Not C3-Knockout
Source: Int J Mol Sci. 2021 Jul 30;22(15):8183. doi: 10.3390/ijms22158183 (PMC8348968; doi:10.3390/ijms22158183)

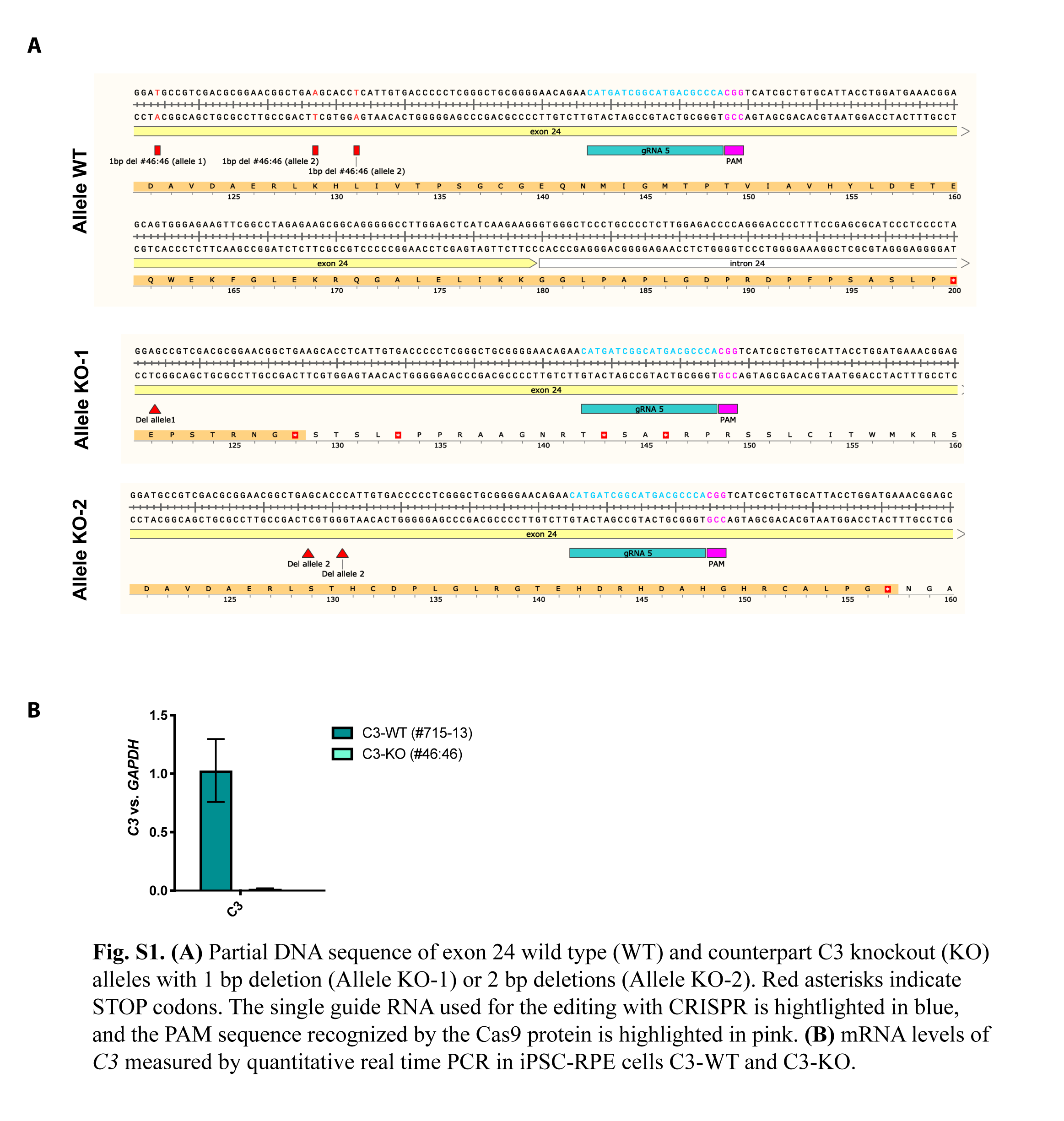

Supplement: Supplementary file 1 [file ijms-22-08183-s001.zip › ijms-1305412-sup/Fig. S1.tif]

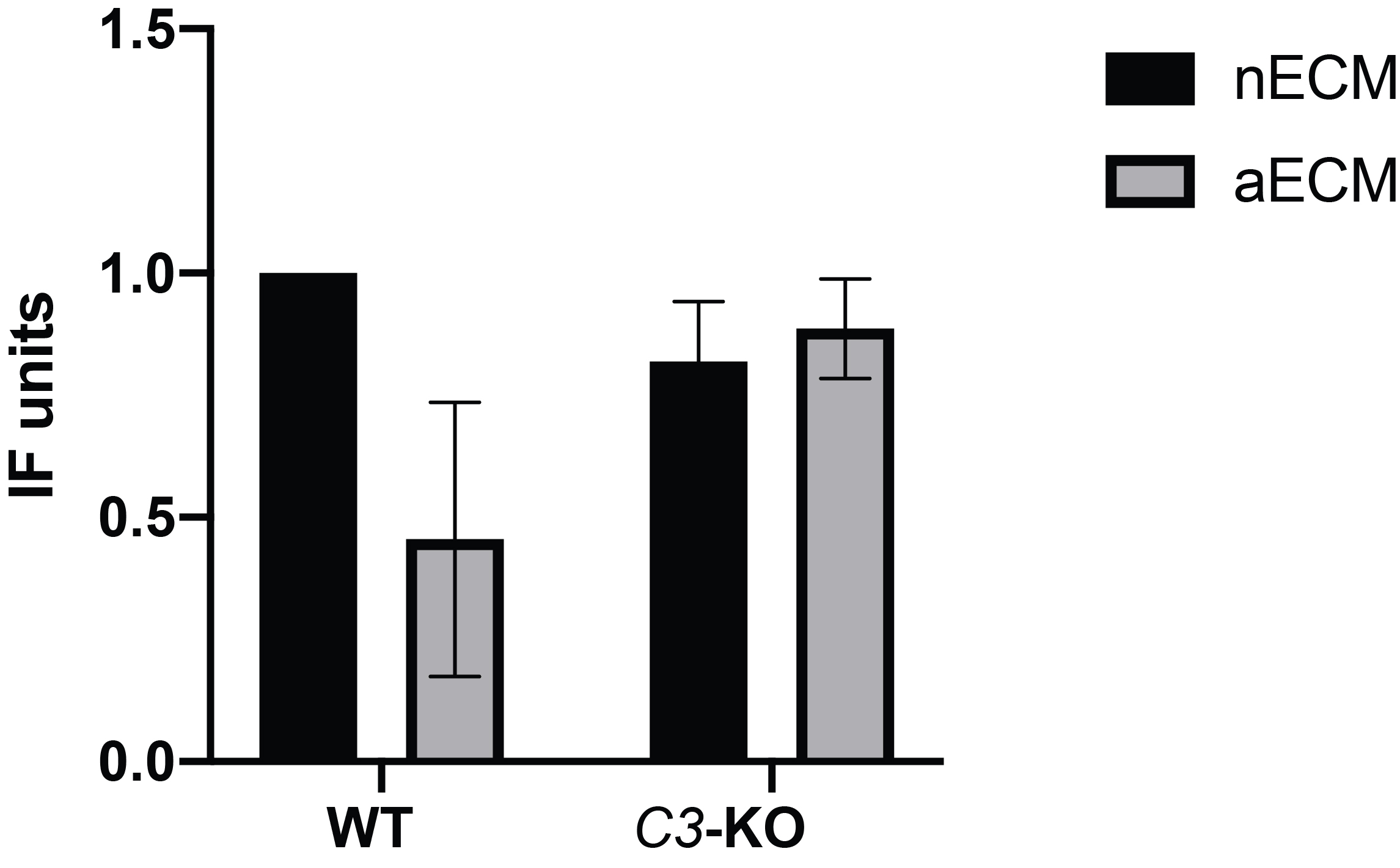

Supplement: Supplementary file 1 [file ijms-22-08183-s001.zip › ijms-1305412-sup/Fig. S2-rev.jpg]

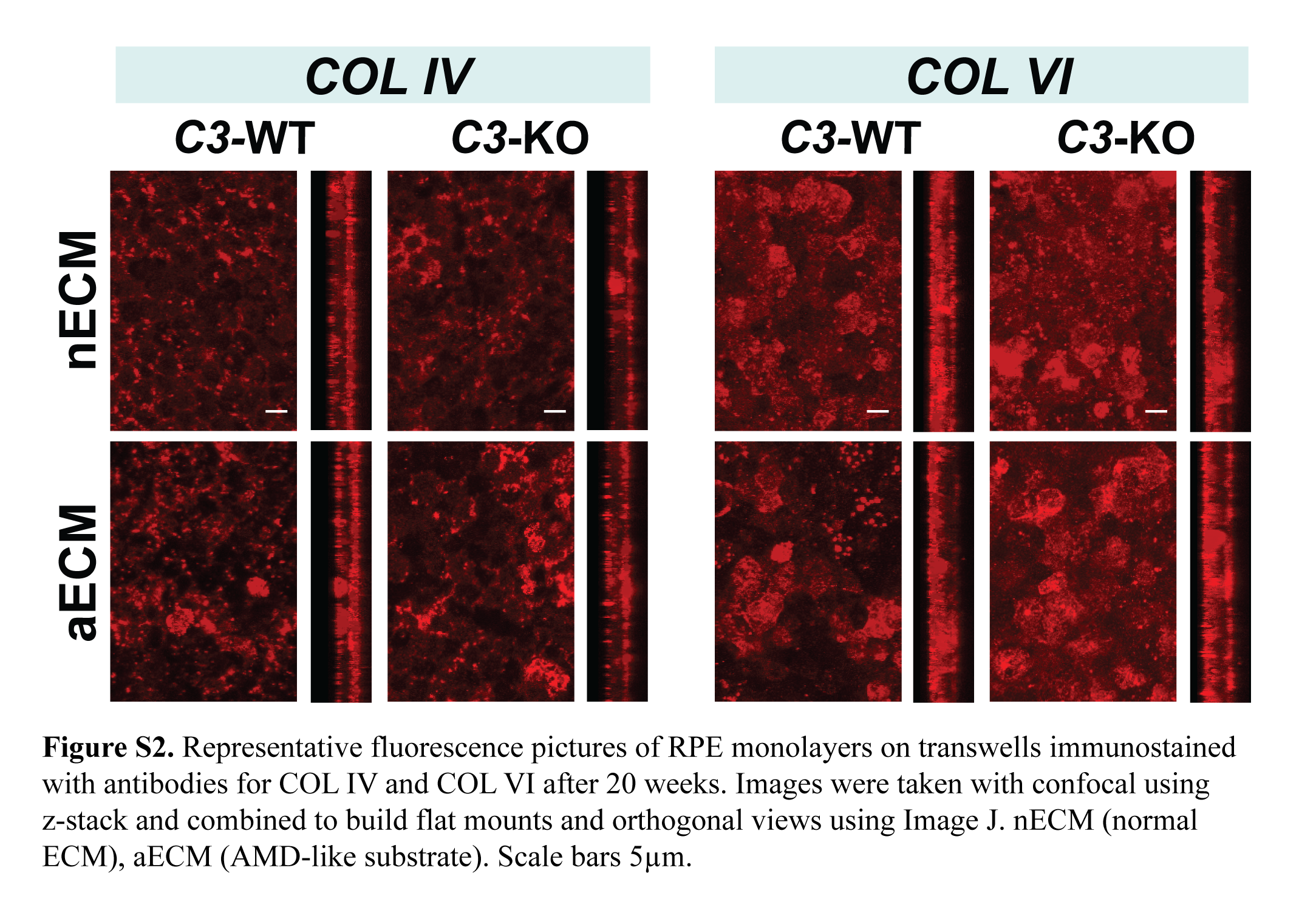

Supplement: Supplementary file 1 [file ijms-22-08183-s001.zip › ijms-1305412-sup/Fig. S3.tif]

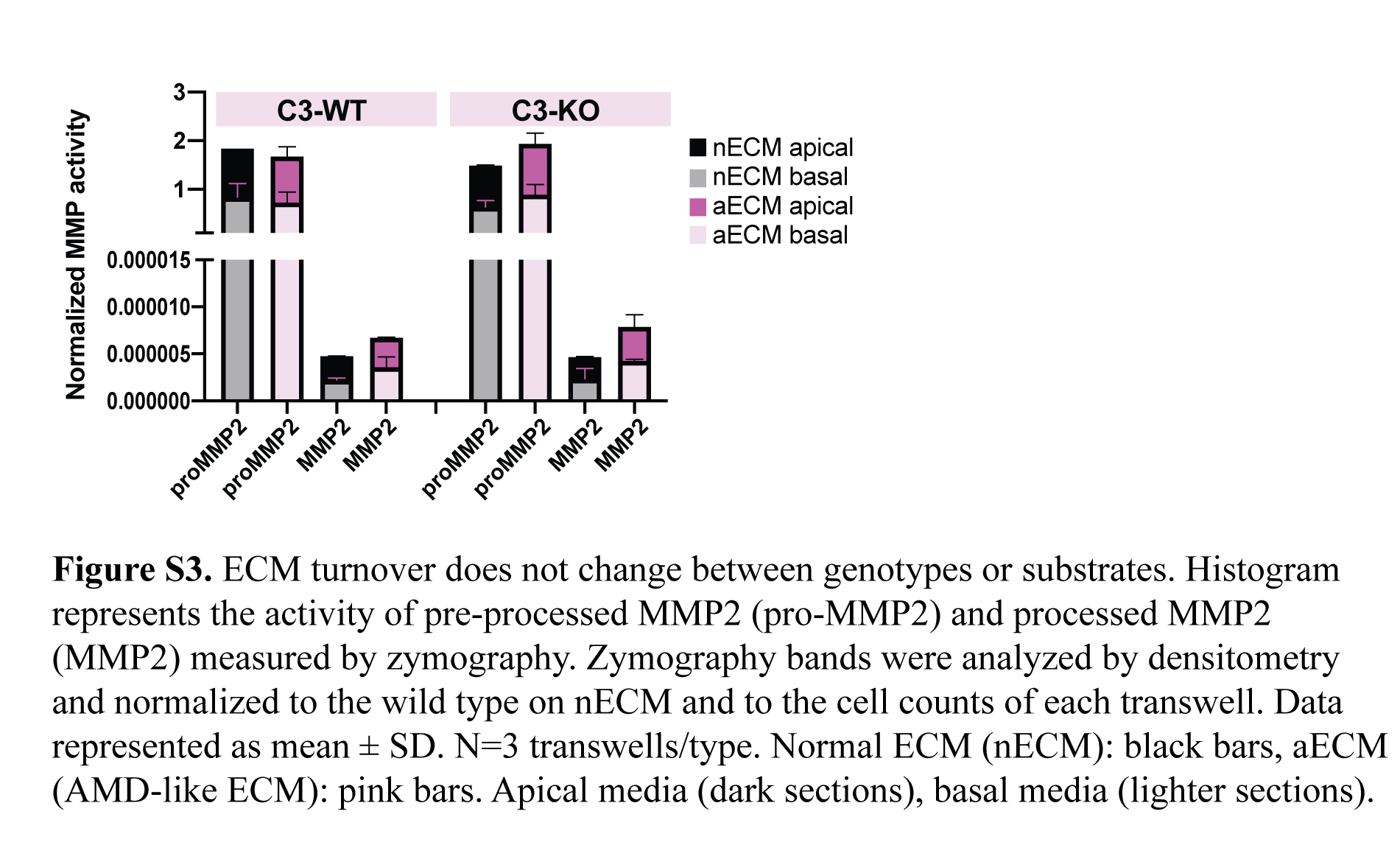

Supplement: Supplementary file 1 [file ijms-22-08183-s001.zip › ijms-1305412-sup/Fig. S4.tif]
